# Assessment of the predictive power the radiation-induced lymphocyte apoptosis method in prostate cancer patients

**DOI:** 10.1038/s41598-024-81450-7

**Published:** 2025-01-09

**Authors:** Gyöngyvér Orsolya Sándor, Gyöngyi Farkas, Gábor Székely, Péter Ágoston, Kliton Jorgo, László Gesztesi, Tibor Major, Csilla Pesznyák, András Herein, Gábor Stelczer, Dalma Mihály, Georgina Fröhlich, Zsolt Jurányi, Zoltán Takácsi-Nagy, Csaba Polgár, Zsuzsa S. Kocsis

**Affiliations:** 1 Department of Radiobiology and Diagnostic Onco-Cytogenetics, Centre of Radiotherapy, National Institute of Oncology, 1122, Ráth György utca 7-9, Budapest, Hungary; 2 National Institute of Oncology, National Tumor Biology Laboratory, Budapest, Hungary; 3 Centre of Radiotherapy, National Institute of Oncology, Budapest, Hungary; 4 Department of Oncology, Semmelweis University, Budapest, Hungary; 5 Department of Biophysics, Eötvös University, Budapest, Hungary

**Keywords:** Cancer, Tumour biomarkers, Urological cancer, Cancer therapy, Radiotherapy, Chromosomes, Apoptosis, Cancer, Cell biology, Oncology, Biomarkers, Predictive markers

## Abstract

Due to the better survival of patients with tumorous diseases, it is increasingly important to predict the side effects of radiotherapy, for which the Radiation-Induced Lymphocyte Apoptosis (RILA) method is proving to be effective in multicentric studies. Prostate cancer is the leading cause of cancer-related deaths among men worldwide, which is usually treated with radiotherapy. We recruited 49 patients with localized prostate cancer and performed RILA measurements before radiotherapy. Patients were treated with CyberKnife (35–40 Gy) or high-dose-rate brachytherapy (1 × 21 Gy). We performed chromosome aberration test for five years and graded side effects, IPSS and QoL questionnaire scores were recorded. We found that RILA% correlated with the IPSS increase (*p* = 0.0016, *r*=-0.44), which was confirmed with negative binomial regression (*p* = 0.0013). The negative predictive value for severe urinary side effects according to the IPSS questionnaire was 87.9% at the lower tertile of RILA values. Chromosome aberrations also correlated with side effects, but when we built models including variables of baseline and treatment characteristics of the patients, RILA, and chromosome aberrations, only RILA predicted a late increase in IPSS score (*p* < 0.0001, adjusted R^2^ = 68%). The RILA method was shown to be predictive of urinary side effects especially of patient-reported outcomes.

**Supplementary Information:**

The online version contains supplementary material available at 10.1038/s41598-024-81450-7.

## Introduction

Prostate cancer stands as one of the prevalent malignancies affecting middle-aged men, emerging as the leading cause of cancer-related deaths among males. Various risk factors, including obesity, advancing age, familial predisposition, ethnic background, and environmental influences, contribute to its onset. From both an epidemiological and genetic perspective, prostate cancer manifests as a heterogeneous disease. Most of them are adenocarcinomas, which have an acinar origin, and androgen dependent tumors^1^. However, in developed countries, the majority of patients present with low or intermediate-risk disease. There are several treatment options available for patients, depending on factors such as the nature of the tumor, PSA levels, grade and stage, as well as the likelihood of recurrence. These may include active surveillance, radiotherapy, hormone therapy, surgery, cryotherapy, and their combinations. 30% of patients receive one type of radiotherapy, which can be divided into two main categories: external beam RT (EBRT), and brachytherapy (BT)^2^. Although radiation therapy is a highly effective treatment, it can have significant side effects that can adversely affect the patients’ quality of life^1,3^. Consequently, in order to select patients susceptible to the development of severe side effects research is needed that addresses the prediction^4^.

Researchers have long been searching for methods to detect radiation-sensitive patients, allowing protocol adjustments before treatment or applying of advanced stereotactic, BT or external adaptive methods to prevent or mitigate side effects. The RILA (Radiation Induced Lymphocyte Apoptosis) method appears to be a promising and simple clinical method for detecting radiation sensitivity^5^, studied in a multicenter study of more than a thousand patients^6^. It is the only technique added to a national guideline (French) as a level I evidence^7^. It is a relatively cheap technique and it can be easily introduced into routine laboratory work and as an easily accessible blood test^8,9^. The method involves examining the number of CD8+ or CD4+ lymphocytes in peripheral blood that undergo apoptosis due to radiation. Patients with low RILA values, indicating a low number of apoptotic cells, are more likely to develop late side effects than patients with high RILA values, who have a higher number of apoptotic lymphocytes. The exact molecular background of this has not yet been discovered, but several theories exist. It was shown, that in patients with low RILA values, lymphocytes tend to become senescent cells after radiation. However, there is a need for a more thorough investigation of the aspects of the mechanism^10^and the link to the genetic background^11,12^.

Several clinical studies proved the efficiency of the RILA method^13^. The most powerful level I evidence is a 10-center study with 502 pathologically verified hormone-negative and positive breast cancer patients^14^. It was found that the median RILA was significantly lower (*p* = 0.004) in women with grade 2 or more severe fibrosis, and no grade 3 fibrosis was observed if RILA was 12% or higher. In a study of 399 patients with various cancer types, those with more severe toxicities had fewer apoptotic cells according to CD8+ and CD4+ RILA. No grade 3 toxicities were observed when CD8+ RILA exceeded 24%^7^. In another study, among 150 breast cancer patients, only those with RILA ≤ 6.9% developed grade ≥ 2 fibrosis^15^. Similarly, in studies involving 50 prostate cancer cases, 79 head and neck cancer patients, and 94 cervical cancer patients, a low RILA rate was found to correlate with increased radiation toxicity^16–18^. In prostate cancer (214 patients), the ability of CD4+ to predict late genitourinary toxicities was confirmed, and a correlation between CD8+ apoptosis rate and overall survival was found^19^.

The disadvantage of the RILA method is that thresholds can vary between patient groups and centers^9^. Therefore, we aimed to establish a threshold for the method, and achieve a clinical validation in prostate cancer patients.

## Patients and methods

### Patients

In our study, we included forty-nine consecutive prostate cancer patients with localized tumor, who had no previous chemo- or radiotherapy. The baseline characteristics are summarized in Supplementary Table 1.

### Radiotherapy

Every patient signed an informed consent and the study was performed according to the Helsinki declarations and approved by the National Ethics Committee (16738-2/2015/EKU).

CyberKnife (Accuray Inc., Sunnyvale, CA, USA) therapy was given with a linear accelerator equipped with a precise robotic arm. Four gold markers were implanted into the prostate two weeks before the planning CT for reproducible target positioning. The total prostate dose was 35–40 Gy (7–8 Gy/fraction). In the case of intermediate and high-risk patients, seminal vesicles were irradiated with 30–32.5 Gy (6–6.5 Gy/fraction) every second workday. These selected high-risk patients did not receive any additional pelvic lymph node irradiation, because of age or comorbidities.

HDR (high-dose-rate) brachytherapy was performed in local anesthesia in a lithotomy position with transrectal ultrasound guidance. After Foley catheter insertion, the prostate was immobilized with two fixation needles, then steel needles were inserted into the prostate. Planning was performed intraoperatively using Oncentra Prostate 3.2.2 (Elekta Brachytherapy, Veenendaal, Netherlands) treatment planning system with HIPO inverse optimization algorithm (Hybrid Inverse Planning Optimization). According to the treatment plan a ^192^Ir source was loaded into the metal needles using a Flexitron afterloader (Elekta Brachytherapy, Veenendaal, Netherlands) and 21 Gy was administered to the whole prostate.

Brachytherapy, was only allowed for prostate with a volume of less than 60 cm^3^ volume and patients with pubic arch interference were excluded. Otherwise, patient preference was taken into account. Fifty consecutive localized prostate cancer patients were enrolled and offered the choice between Cyberknife and HDR-BT. One patient has been excluded, because RILA samples were damaged during shipment. Subgroup analysis in the HDR-BT treated group was not feasible, because of the low sample size.

Volumes irradiated with given doses were collected from the radiotherapy planning systems, because they can affect side effects they were used as possible predictors.

Blood samples were collected from prostate cancer patients before radiotherapy, at the end of the treatment, and 3, 6, 9, 12, 24, 36, 48, and 60 months after treatment completion. At these time points, side effects were assessed by physicians and by the questionnaires (International Prostate Symptom Score - IPSS and Quality of Life - QoL).

### RILA

For RILA measurements, we mixed the blood taken before therapy with tenfold cell culture media (RPMI (Gibco, cat.: 21875091) with 20% FBS (Cytiva HyClone, cat.: SV30160.03) medium and cultured it for 24 h at 37 °C. The next day it was irradiated with a total dose of 8 Gy (6 MV photon, 100 MU/minute). Two days later, lymphocytes were labelled with 10 µl CD8-FITC antibody (eBioscience, cat.: 555366) in 200 µl suspension for 15 min. After 2 × 20 min of erythrolysis (BD, cat.: 349202), the samples were treated with propidium iodide (10 µg/ml eBioscience, cat.: P4864) and RNase (100 µg/ml Sigma-Aldrich, cat.: R6513), followed by a flow cytometry analysis (BD FACS Canto II, FACS Diva software). The apoptotic cell population was determined according to shrinkage and DNA loss (on the propidium iodide scale). The samples were measured in duplicates for 8 Gy and 0 Gy. RILA value is the mean of the apoptotic CD8+ cells percentage after 8 Gy minus the mean of the apoptotic CD8+ cells in the non-irradiated samples. Preparation times were recorded: storage time is the time between blood draw and mix with culture media; pre-irradiation time is between mix with media and irradiation; post-irradiation incubation time is the time between irradiation and lysis, FACS lag is the time between lysis and FACS measurement.

### Chromosome aberrations

Blood samples (800 µl) were cultured in RPMI media (9 ml) supplemented with 15% FBS, and Penicillin + Streptomycin, and stimulated with Phytohemagglutinin M (2 v/v%, Gibco, cat: 10576015). After 48 h the microtubules were inhibited with Colcemid (0.1 µg/ml, Gibco, cat: 15212012) for two hours. Hypotonic treatment was performed (0.075 M KCl) for 10 min at 37^o^C and the metaphase chromosomes were fixed 4–5 times with cold 3:1 methanol: acetic acid. Smears on glass slides were stained with Giemsa solution and analyzed on 1000x magnification. The samples were evaluated on four slides (25–25 metaphase). Chromatid-type aberrations (chromatid break and exchange), and chromosome type aberrations (chromosome break, dicentric, ring, translocation) were registered and summed as total aberration values. The aberrant cell value is the count of cells with any aberrations.

### Variables

RTOG (Radiation Therapy Oncology Group) - EORTC (European Organisation for Research and Treatment of Cancer) grades were given blindly to the RILA and chromosome aberration results. The acute cumulative side effects were scored according to the grade directly after therapy and 3 months later. The late side effects were also monitored in a cumulative manner starting at 6 months after therapy.

Patients answered the IPSS and QoL questionnaires before therapy and at the same follow-up points as the graded side effects were recorded. In both questionnaires, a higher score means more side effects and a lower quality of life. To eliminate the differences in scores before therapy between the patients, increases in score were calculated: the baseline values were subtracted from the maximal cumulative acute and late scores. On the other hand, absolute, acute and late scores were used when categorization of the IPSS score was needed (mild, moderate, severe), because there is no internationally accepted categorization of the IPSS score increase. In the case of Chi-squared tests, ROC analysis and in part of the multivariate regression analyses absolute cumulative scores were used.

An increase of PSA with 2 ng/ml compared to nadir PSA was considered a biochemical relapse. Failure event was defined (failure-free survival) as either biochemical failure or clinical relapse event at the first occurrence. Grade 2 ≤ GU side effect plus failure free combined survival and 20 ≤ IPSS score plus failure-free survival were also analyzed.

### Statistical analysis

Tertiles were formed around the 0.33 percentiles of RILA values recommended by others^9,14^. Spearman rank order correlation analysis was used to test the connection between RILA, time and chromosome aberration parameters, because RILA is not normally distributed (Shapiro-Wilk test *p* < 0.0126). Chi-squared tests were applied to compare grades of side effects between RILA tertiles. Negative binomial regression was applied to the test if the RILA value affects side effects. Receiver operating characteristic (ROC) curve analyses were applied to test the predictive capacity of RILA, sensitivity, specificity, positive and negative predicted values were reported. Youden index was calculated for cut point determination. Multivariate non-linear regression analyses with forward stepwise model building were performed to test the effect of RILA, baseline and treatment variables on side effects. Cox regression was applied to test if RILA affects the survival including severe side-effect-free survivals. StatSoft STATISTICA 12, GraphPad Prism (San Diego, CA, USA) and IBM SPSS Statistics 25 were applied for statistical tests.

## Results

The median follow-up of our 49 patients was 48 (min 24, max 60) months. The average RILA value was 14.3 ± 9.3% the lower tertile was at 5.8%, the upper tertile 18.9%. The mean RILA value was 13.0 ± 8.4% in case of patients treated by CyberKnife and 17.7 ± 10.8% in the case of HDR-BT patients.

## Setting up RILA

The mean value of the measurements of the samples irradiated with 8 Gy significantly correlated (Spearman *r* = 0.87 for the whole group, *r* = 0.92 for CyberKnife group) with the RILA value, but the un-irradiated values did not.

We analyzed the dependence of RILA values from preparation times and found no significant correlations. The correlation (Spearman) coefficients were: 0.1190 for storage time (4.6 ± 1.9 h), 0.0427 for pre-irradiation time (22.3 ± 0.2 h), −0.2586 for post-irradiation incubation time (48.8 ± 0.6 h), −0.1377 for FACS lag (1.7 ± 0.3 h), 0.0239 for total preparation time (77.4 ± 2.0 h). There were no significant correlations between time variables and RILA% in the CyberKnife group either.

Neither smoking, age, d’Amico prostate cancer risk, nor hormone treatment had a significant effect on RILA values in either group (Spearman correlation coefficients for the whole cohort: −0.0495, −0.2582, −0.0574, −0.0117, respectively).

### RILA and side effects

Grade 2 acute genitourinary (GU) and gastrointestinal (GI) side effects were observed in 51%, and in 2% respectively. The frequency of grade 2 or higher late GU and GI toxicities was 22.5% and 0%, respectively. Grade 3 late GU side effects occurred only in 4.1%. There was no grade 3 late GI side effect observed. According to the IPSS score, severe acute and late urinary complaint occurred in 6.1% and 14.3% respectively (Table 1).

**Table 1:** Acute and late side effects according to EORTC-RTOG grades and IPSS score.

	Acute GU side effects			Acute GI side effects			Cumulative late GU side effects			Cumulative late GI side effects		
	Whole cohort	Cyberknife treated group	p	Whole cohort	Cyberknife treated group	p	Whole cohort	Cyberknife treated group	p	Whole cohort	Cyberknife treated group	p
grade 0	16 (32.7%)	5 (14.3%)	0.1465	40 (81.6%)	26 (74.3%)	0.7209	24 (49.0%)	16 (45.7%)	0.9794	44 (89.8%)	30 (85.7%)	0.5690
grade 1	8 (16.3%)	6 (17.1%)		8 (16.3%)	8 (22.9%)		14 (28.6%)	10 (28.6%)		5 (10.2%)	5 (14.3%)	
grade 2	25 (51.0%)	24 (68.6%)		1 (2.0%)	1 (2.9%)		9 (18.4%)	7 (20.0)		0	0	
grade 3	0	0		0	0		2 (4.1%)	2 (5.7%)		0	0	

	Acute IPSS score						Cumulative late IPSS score					
	Whole cohort	Cyberknife treated group	p				Whole cohort	Cyberknife treated group	p			
mild (0–7)	35 (71.4%)	23 (65.7%)	0.6744				14 (28.6%)	7 (20.0%)	0.8334			
moderate (8–19)	10 (20.4%)	7 (20.0%)					28 (57.1%)	22 (62.9%)				
severe (20–35)	3 (6.1%)	4 (11.4%)					7 (14.3%)	6 (17.1%)				

(GU: genitourinary side effects, GI: gastrointestinal side effects, IPSS: international prostate symptom score).

We tested the correlation between RILA values and genitourinary (GU), gastrointestinal (GI) side effects and patient-reported outcome measures. In the case of IPSS and QoL scores, we subtracted the baseline value from the maximal, cumulative score. There was no significant correlation between RILA and acute toxicities. Also, no significant correlation was found between late, graded side effects and RILA values. However, cumulative, late IPSS increase and late absolute IPSS correlated with RILA values (*r*=−0.44 *p* = 0.0015 and *r*=−0.38 *p* = 0.0065, respectively). We made a subgroup analysis of CyberKnife -treated patients (the sample size is low in the HDR-BT treated group), as radiotherapy type can affect side effects. We found that the late IPSS and RILA value correlated (*r*=−0.47 *p* = 0.0046) in this group as well.

There was no difference in cumulative graded GU side effects between the tertiles of RILA% with the Chi-square test (*p* = 0.0826), but, in the case of IPSS (mild, moderate and severe) the difference was significant (*p* = 0.0367). We found no significant differences in the CyberKnife group (*p* = 0.4369 for graded GU side effects and *p* = 0.0573 for IPSS).

In the negative binomial regression analysis, late, cumulative IPSS increase was predicted by RILA% (goodness of fit Omnibus test *p* = 0.0013), but we did not find any predictive models with graded acute or late toxicities. In the CyberKnife group in the only significant model RILA% also predicted a late increase of IPSS (*p* = 0.0146).

Receiver operating characteristic analysis was performed to assess the performance of the RILA method. The area under the curve (AUC) was 0.644 (CI:0.476–0.811) in the case of late GU toxicities and 0.660 (CI: 0.509–0.811) in the case of IPSS. In this analysis we used the absolute score as there are no internationally accepted cut-off values for IPSS increase. Youden index was calculated and to maximize both sensitivity and specificity, it was the highest at 15.95 RILA%.

We report here the sensitivity and specificity at the lower tertile (5.76% of RILA) and the upper tertile (18.92% RILA) and the values at the maximal Youden index (Table 2).

**Table 2:** Sensitivity and specificity values of different RILA cut-off values.

		GU				IPSS			
	RILA value (%)	Sensitivity	Specificity	PPV	NPV	Sensitivity	Specificity	PPV	NPV
lower tertile	5.76	54.6	73.7	37.5	84.8	42.8	69.1	18.8	87.9
upper tertile	18.92	81.8	39.5	27.3	87.5	100.0	40.5	21.2	100
max. Youden	15.95	81.8	52.6	33.3	90.9	100.0	52.4	25.9	100

(GU: genitourinary side effects, IPSS: international prostate symptom score, PPV: positive predictive value, NPV: negative predictive value).

In the group treated with the CyberKnife, the AUC was 0.530 (CI:0.327–0.733) and 0.569 (CI:0.388–0.750) for genitourinary side effects and IPSS scores, respectively (Supplementary Table 2).

### Chromosome aberrations and side effects

Chromosome aberrations are suggested to be predictive markers of toxicities and we found significant correlations (Table 3). Aberrations directly after radiotherapy showed the most significant associations. Chromosome breaks and total aberrations appeared in the most significant correlations. Furthermore, there was some correlation between aberrations and RILA values (chromosome breaks 3 and 36 months after radiotherapy; *p* = 0.0207; *p* = 0.0148, respectively) as well.

**Table 3:** Significant correlations between chromosome aberrations, side effects, and RILA values over time.

	Before therapy	Directly after therapy	3 months	6 months	9 months	12 months	24 months	36 months	48 months	60 months
Acute GU side effects		chrom. br., dic., total a., ab.cell	dic.			dic.				
Acute GI side effects		chrom. br.			total a.,ab. cell,		chrom. br.		chrom. br., ab.cell	
Cumulative late GU side effects										total a., ab.cell
Cumulative late GI side effects										
Acute IPSS score		dic., total a., ab.cell								
Acute QoL score		chrom. br.					dic., total a., ab.cell			
Late cumulative IPSS increase			chrom. br.							
Late cumulative QoL increase		total a.								
Late cumulative IPSS score	chrom. br., total a., ab.cell	chrom. br., total a.	chrom. br.	dic., ab.cell		dic.		total a., ab.cell		
Late cumulative QoL score		chrom. br., total a.		dic.						
RILA value (%)			**chrom. br.**					**chrom. br.**		

(GU: genitourinary side effects, GI: gastrointestinal side effects, IPSS: international prostate symptom score, QoL: quality of life, chrom. br.: chromatid breaks, total a.: total aberrations, dic.: dicentric + ring chromosomes, ab. Cell: aberrant cells).

There are eight correlations between the chromosome aberrations and side effects in the smaller CyberKnife group. Furthermore, the RILA value correlated with chromosome breaks three months after radiotherapy (Supplementary Table 3).

### Multivariate prediction of side effects

Multivariate regression models were built to interpret late cumulative GU side effects and IPSS, QoL absolute scores and increases (Table 3), the effect of the baseline characteristics of the patients (age, smoking status, prostate cancer risk), the administered treatments (hormone therapy, radiotherapy type and irradiated volume) and RILA%. As the RILA value correlated with chromosome breaks, to decrease collinearity, we decided to use total aberrations in further models, which has the second highest number of correlations with side effects. We introduced the total aberrations directly after radiotherapy into the multivariate models because this time point was the most tightly associated with the side effects, and chromosome aberrations were initiated to forward stepwise model building. V_10Gy_ as volume was chosen, because it showed the strongest correlation with side effects in prior analysis (Spearman correlation coefficient *r* = 0.20, 0.25, 0.24 for V_10%_, V_10Gy_, and V_100%_ respectively). The criteria to enter was *p* < 0.05. We summarized the significant models in Table 4.

Acute and late, graded gastrointestinal side effects were predicted by smoking and in case of acute toxicities, by type of radiotherapy. Late IPSS increase was dependent on RILA%, smoking, radiotherapy type and initial IPSS. Near 45% of the variance of the IPSS increase between the patients was explainable with these variables (adjusted R^2^). Smoking was a predictor in the case of graded GU side effects and IPSS increase. Hormone therapy decreased the late QoL scores and late QoL scores minus baseline scores. CyberKnife therapy caused more side effects and higher quality of life score (lower quality) in the significant model of acute GU side effects, late QoL score, acute and late QoL increase and late IPSS increase and score. Initial questionnaire scores (the baseline conditions of the patients) were significant predictors of late questionnaire score changes. There was no significant model for gastrointestinal side effects.

As treatment type had a big impact on side effects, we analyzed the subgroup who received CyberKnife therapy (Supplementary Table 4). The acute IPSS and QoL score has no significant model anymore. The predictors have mostly remained the same. But in the case of the late IPSS questionnaire score, smoking arose as a new predictor. Furthermore, in this group, age, prostate cancer risk and V_10Gy_ volume predicted cumulative late GU side effects.

**Table 4:** Multivariate regression models of side effects.

	Acute GU side effects	Cumulative late GU side effects	Acute IPSS score	Acute QoL score	Late cumulative IPSS increase	Late cumulative QoL increase	Late cumulative IPSS score	Late cumulative QoL score
Model p	< 0.0001	0.010	0.002	0.008	< 0.001	0.002	0.001	0.005
Adjusted R^2^ (%)	46.3	14.9	22.8	16.1	44.9	30.7	25.3	22.7
RILA value (%)					0.014			
Smoking (yes/no)	0.044	0.010			0.003			
Hormone therapy (yes/no)						0.048		0.035
Age								
Risk (d’Amico)								
Radiotherapy (Cyberknife /HDR)	< 0.0001			0.008	0.008	0.020	0.001	0.038
Initial questionnaire score					< 0.001	0.002		
V_10Gy_ (cm^3^)			0.002					
Total aberrations (directly after RT)								

Baseline characteristics of the patients, treatments, RILA, and chromosome aberrations were introduced to the model. (GU: genitourinary side effects, GI: gastrointestinal side effects, IPSS: international prostate symptom score, QoL: quality of life, HDR: high dose rate Brachytherapy, RT: radiotherapy).

### Survival and RILA

Cox regression analyses were performed to asses RILA’s contribution to survival variables. Besides conventional variables like biochemical relapse-free survival, local relapse-free survival, regional relapse-free survival, disease-free survival, failure-free survival, overall survival; side effect-free survival times were monitored. RILA did not show any connection with any of the conventional survivals; grade 2 or higher GU side effect-free survival or severe IPSS symptom-free survival. If we combined side effects and failure-free survival, there was no significant regression model either. Due to the low event number the association of RILA and survival variables is not feasible in the group treated with CyberKnife.

## Discussion

In our study, we analyzed the RILA value and side effects of 49 prostate cancer patients. We performed all the tests recommended before, but the low number of patients is clearly a limitation of our study. However, we could show the predictive power of the RILA method. We found more significant models in the case of patient-reported outcomes than graded side effects. We suggest that the limited scale and variance of the grades can limit the analysis. Furthermore, questionnaires exclude bias of the healthcare professionals. Patient-reported outcomes were also found to be more sensitive in detecting toxicity after radiotherapy than RTOG or CTCAE scaling in the large-scale multicenter study of prostate SBRT by Tree et al.^20^.

In the mixed cohort of Ozsahin et al., where 36 prostate cancer patients were included, CD8+ RILA resulted in AUC of 0.83, but they did not perform any subgroup analysis^8^. Later, in a study by Talbot et al. 471 prostate cancer patients had significantly higher RILA values than lung or breast cancer patients, highlighting a need for determination of the cut values of this group separately^9^. On the other hand, in a study of 88 prostate patients, there was no significant difference in RILA value between GU grade 1 and grade 2 or more. The toxicity in their group was as low as in our center, and they did not aim to analyze patient questionnaires^21^. In an accidentally overdosed prostate cancer group (*n*= 245) there was also no association between RILA and grade 2 or higher grade toxicities in multivariate analysis. Here the side effects were also graded, the RILA was measured 6 months after the radiotherapy and they suggested that this overdose exceeded the limit of individual radiosensitivity and overdosage determined the toxicities^22^. However, in 218 prostate patients hazard ratio of GU toxicities was significantly higher in the case of low CD4+ RILA value (measured with Annexin V)^19^. We hypothesize that besides group size, both the methodology of recording the side effects and RILA measurement technology influenced the detection of the association.

We observed the area under the curve (0.660), positive and negative predictive values similar to those published in a larger breast cancer cohort. Azria et al. observed 0.62 AUC, the sensitivity was 34% and the specificity 67% at their lower tertile RILA value and ours were 43% and 69% for IPSS score, respectively. The RILA is a negative predictor, which is mirrored in the negative predictive value, which was 87.9% in our cohort. In contrast, the positive predictive value was 21.2% at the upper tertile. In the French trial, the positive predictive value (PPV) was 22% at the lower tertile and the negative predictive value (NPV) was 91% at the upper tertile cut point. In the Ozsahin et al. cohort AUC was greater (0.827) and they recorded a particularly high PPV of 83% at the lower tertile and the NPV was 86% at the upper tertile^8^. As mentioned above, a higher negative predictive value was observed in the case of the IPSS score than in the case of graded GU toxicities.

The choice of the cut point of RILA value is also an important matter. We reported the values at the lower and upper tertile as shown before^8,9,14^ and at the Youden index point as well. Laboratories may benefit from starting clinical implementation of the RILA method, by measuring approximately 50 patients, to determine their tertile points. They can choose the upper tertile cut point, if they want higher sensitivity, because they may have a high capacity to treat patients with adaptive radiotherapy, brachytherapy, proton therapy, or other specialized modalities. Or they can choose the lower tertile cut point, if they do not have a high capacity to treat all the possibly radiosensitive patients cautiously, and want a more specific cut point. Later, when the center collects the side effect data, the Youden index or other cut point calculation method can be applied.

To facilitate the clinical introduction of the RILA method, we analyzed the effect of preparation times. We could not find any correlation between RILA values and preparation times in contrast with other studies. We hope, that our low variance of time intervals was enough to eliminate this additional factor. However, the RILA value can be adjusted with the regression beta coefficients of preparation time variables. The clinical application tried to be facilitated by commercialization as well, and a company (NovaGray) is capable of diffusing the test with the legal recommendations of CE marks. They also applied radiomimetic drugs to substitute radiation to enable the performance of RILA method without irradiator and radiotherapy centers^23^.

RILA was no predictor of any of the survival variables we analyzed. However, the association was shown in rectal^24^and cervix^25^ tumor patients. As the molecular mechanism of RILA is not revealed yet, it is possible, that it is a predictor of tumor relapse as well. The localized prostate cancer patients of our cohort had a very good prognosis, it is possible, that the low number of events prevented the connection from being found.

Chromosome aberrations are proposed to be predictive markers of radiogenic side effects^26–28^. We analyzed the correlations with side effects and found significant associations, but it was rare. The RILA value also correlated with chromosome breaks (at 3 and 36 months) (Table 3). However, beside other predictors, total aberrations (directly after the last fraction) were not independent predictors. Chromosome aberration technique is robust and cheap, but highly labor-intensive technique. RILA assay might be easier to apply in clinical practice.

CyberKnife is a cutting-edge technique of radiotherapy. Its precision allows therapists to shrink the safety margins between CTV (clinical target volume) and PTV (planning target volume). It is also a non-invasive technique compared to brachytherapy and only 5 fractions are needed in the case of prostate therapy. However, stereotactic hypofractionation is still more commonly performed with linear accelerators. Yoshioka et al. presented a dosimetric comparison between robotic and conventional SBRT and HDR therapy. We observed similar coverage of the PTV as they presented. 95.6 ± 0.5% V_100%_ was observed in Yoshhioka et al. and 95.8 ± 0.8% in our study for Cyberknife vs. 96.7 ± 1.4% in Yoshioka et al. and 98.3 ± 0.9% V_100%_ was found in our study for HDR patients. Both coverages of HDR treatments were higher (*p* < 0.01 and *p* < 0.0001). In comparison, the V_100%_ of the conventional LINAC based SBRT was lower in their cohort than V_100%_of the SBRT robotic arm^29^. These differences in coverage, hot spots in the PTV and irradiation time could affect our chromosomal aberration results, but do not affect RILA, decreasing correlation between the two.

We have not registered more than Grade 2 gastrointestinal side effects. The cause can be the small cohort size and that the Cyberknife therapy using marker tracking guided by the three gold markers placed into the prostate allows the use of narrow margins and provides good rectum protection. In the case of HDR brachytherapy, the intraoperative dose planning is quasi-real time and CTV is equal to PTV, and the steep dose gradient around the source helps to prevent damage to healthy tissues. There was also no grade 3 or higher RTOG toxicity in the PACE-B trial and only 2% grade 2 side effect was observed in 414 patients two years after SBRT^20^. After 30 month follow-up (min. 12 months), only 2.9% of grade 2 (and no grade 3) late rectal toxicity were found in 304 prostate SBRT patients after 35–36.2 Gy doses^30^.

We had enough patients for a subgroup analysis of CyberKnife therapy patients and obtained very similar results to the whole population. Type of therapy was a significant predictor of side effects besides RILA values in the multivariant analyses. Smoking, hormone therapy and irradiated volume also affected some of the side effects. However, age and prostate cancer risk were not predictors in the whole cohort. The adjusted regression coefficient changed on a wide scale but it did not exceed 70%. We believe that the doses of the organs at risk, the hotspots mentioned above, etc. in the personalized radiotherapy plan can explain the rest of the variance of the side effects. Normal Tissue Complication Probability (NTCP) models on big cohorts to describe the contribution of the different organ doses are needed.

## Conclusion

We found the RILA method to be a predictive marker of urinary side effects. The patient questionnaire scores showed a better association with RILA values than graded side effects, therefore they may be more feasible for these studies. Chromosome aberrations correlated with side effects but were not shown to be independent predictors. Our study helps to prove the feasibility of RILA assay in prostate cancer patients and therefore it can help to improve the quality of life of this large patient group.

## Electronic supplementary material

Below is the link to the electronic supplementary material.

Supplementary Material 1

## Data Availability

The datasets generated during and/or analysed during the current study are available from the corresponding author on reasonable request.

## References

[1] Wasim, S., Lee, S. Y. & Kim, J. Complexities of prostate cancer. *Int. J. Mol. Sci.* **23**. 10.3390/ijms232214257 (2022). 10.3390/ijms232214257 PMC9696501 36430730

[2] Gordon, L., Dickinson, A. & Offredy, M. Information in radiotherapy for men with localised prostate cancer: An integrative review. *Eur. J. Cancer Care (Engl)*. **28**, e13085. 10.1111/ecc.13085 (2019). 31066129 10.1111/ecc.13085

[3] Sekhoacha, M. et al. Prostate cancer review: Genetics, diagnosis, treatment options, and alternative approaches. *Molecules* **27**. 10.3390/molecules27175730 (2022). 10.3390/molecules27175730 PMC9457814 36080493

[4] Dilalla, V., Chaput, G., Williams, T. & Sultanem, K. Radiotherapy side effects: Integrating a survivorship clinical lens to better serve patients. *Curr. Oncol.* **27**, 107–112. 10.3747/co.27.6233 (2020). 32489253 10.3747/co.27.6233 PMC7253739

[5] Azria, D. et al. Data-based radiation oncology: Design of clinical trials in the toxicity biomarkers era. *Front. Oncol.* **7**, 83. 10.3389/fonc.2017.00083 (2017). 28497027 10.3389/fonc.2017.00083 PMC5406456

[6] Seibold, P. et al. REQUITE: A prospective multicentre cohort study of patients undergoing radiotherapy for breast, lung or prostate cancer. *Radiother Oncol.* **138**, 59–67. 10.1016/j.radonc.2019.04.034 (2019). 31146072 10.1016/j.radonc.2019.04.034

[7] Lapierre, A. et al. Tumour and normal tissue radiosensitivity. *Cancer Radiother*. **26**, 96–103. 10.1016/j.canrad.2021.11.008 (2022). 34953704 10.1016/j.canrad.2021.11.008

[8] Ozsahin, M. et al. CD4 and CD8 T-lymphocyte apoptosis can predict radiation-induced late toxicity: A prospective study in 399 patients. *Clin. Cancer Res.* **11**, 7426–7433. 10.1158/1078-0432.CCR-04-2634 (2005). 16243816 10.1158/1078-0432.CCR-04-2634

[9] Talbot, C. J. et al. Multi-centre technical evaluation of the radiation-induced lymphocyte apoptosis assay as a predictive test for radiotherapy toxicity. *Clin. Transl Radiat. Oncol.* **18**, 1–8. 10.1016/j.ctro.2019.06.001 (2019). 31341970 10.1016/j.ctro.2019.06.001 PMC6610684

[10] Lapierre, A. et al. Improving patients’ life quality after radiotherapy treatment by predicting late toxicities. *Cancers (Basel)*. **14**. 10.3390/cancers14092097 (2022). 10.3390/cancers14092097 PMC9099838 35565227

[11] Fuentes-Raspall, M. J. et al. Apoptosis for prediction of radiotherapy late toxicity: Lymphocyte subset sensitivity and potential effect of TP53 Arg72Pro polymorphism. *Apoptosis* **20**, 371–382. 10.1007/s10495-014-1056-2 (2015). 25398538 10.1007/s10495-014-1056-2

[12] Azria, D. et al. Single nucleotide polymorphisms, apoptosis, and the development of severe late adverse effects after radiotherapy. *Clin. Cancer Res.* **14**, 6284–6288. 10.1158/1078-0432.CCR-08-0700 (2008). 18829510 10.1158/1078-0432.CCR-08-0700 PMC2771757

[13] Fhoghlu, M. N. & Barrett, S. A. Review of radiation-induced lymphocyte apoptosis as a predictor of late toxicity after breast Radiotherapy. *J. Med. Imaging Radiat. Sci.* **50**, 337–344. 10.1016/j.jmir.2019.02.004 (2019). 31176443 10.1016/j.jmir.2019.02.004

[14] Azria, D. et al. Radiation-induced CD8 T-lymphocyte apoptosis as a predictor of breast fibrosis after radiotherapy: Results of the Prospective Multicenter French Trial. *EBioMedicine* **2**, 1965–1973. 10.1016/j.ebiom.2015.10.024 (2015). 26844275 10.1016/j.ebiom.2015.10.024 PMC4703704

[15] Bourgier, C. et al. Concurrent or sequential letrozole with adjuvant breast radiotherapy: Final results of the CO-HO-RT phase II randomized trial. *Ann. Oncol.* **27**, 474–480. 10.1093/annonc/mdv602 (2016). 26681684 10.1093/annonc/mdv602 PMC4907345

[16] Pinkawa, M., Brzozowska, K., Kriehuber, R., Eble, M. J. & Schmitz, S. Prediction of radiation-induced toxicity by in vitro radiosensitivity of lymphocytes in prostate cancer patients. *Future Oncol.* **12**, 617–624. 10.2217/fon.15.334 (2016). 26806671 10.2217/fon.15.334

[17] Bordon, E. et al. Prediction of clinical toxicity in locally advanced head and neck cancer patients by radio-induced apoptosis in peripheral blood lymphocytes (PBLs). *Radiat. Oncol.* **5**(4). 10.1186/1748-717X-5-4 (2010). 10.1186/1748-717X-5-4 PMC2827476 20109191

[18] Bordon, E. et al. Role of CD4 and CD8 T-lymphocytes, B-lymphocytes and natural killer cells in the prediction of radiation-induced late toxicity in cervical cancer patients. *Int. J. Radiat. Biol.* **87**, 424–431. 10.3109/09553002.2010.537433 (2011). 21142701 10.3109/09553002.2010.537433

[19] Foro, P. et al. Relationship between radiation-induced apoptosis of T lymphocytes and chronic toxicity in patients with prostate cancer treated by radiation therapy: A prospective study. *Int. J. Radiat. Oncol. Biol. Phys.* **88**, 1057–1063. 10.1016/j.ijrobp.2014.01.002 (2014). 24661659 10.1016/j.ijrobp.2014.01.002

[20] Tree, A. C. et al. Intensity-modulated radiotherapy versus stereotactic body radiotherapy for prostate cancer (PACE-B): 2-year toxicity results from an open-label, randomised, phase 3, non-inferiority trial. *Lancet Oncol.* **23**, 1308–1320. 10.1016/S1470-2045(22)00517-4 (2022). 36113498 10.1016/S1470-2045(22)00517-4

[21] Malisic, E. et al. Association of polymorphisms in TGFB1, XRCC1, XRCC3 genes and CD8 T-lymphocyte apoptosis with adverse effect of radiotherapy for prostate cancer. *Sci. Rep.* **12**, 21306. 10.1038/s41598-022-25328-6 (2022). 36494413 10.1038/s41598-022-25328-6 PMC9734114

[22] Vogin, G. et al. Absence of correlation between radiation-induced CD8 T-lymphocyte apoptosis and sequelae in patients with prostate cancer accidentally overexposed to radiation. *Oncotarget* **9**, 32680–32689. 10.18632/oncotarget.26001 (2018). 30220974 10.18632/oncotarget.26001 PMC6135683

[23] Azria, D. et al. Radiation-induced lymphocyte apoptosis assay: Primetime for routine clinical use? *Cancer Radiother*. **28**, 442–448. 10.1016/j.canrad.2024.06.002 (2024). 39304399 10.1016/j.canrad.2024.06.002

[24] Winkler, S. et al. Ex vivo apoptosis in CD8 + lymphocytes predicts rectal cancer patient outcome. *Gastroenterol Res Pract* **2016**, 5076542. 10.1155/2016/5076542 (2016). 27340400 10.1155/2016/5076542 PMC4908238

[25] Ordonez, R. et al. Radio-induced apoptosis of peripheral blood CD8 T lymphocytes is a novel prognostic factor for survival in cervical carcinoma patients. *Strahlenther Onkol*. **190**, 210–216. 10.1007/s00066-013-0488-x (2014). 24362501 10.1007/s00066-013-0488-x

[26] Borgmann, K. et al. Individual radiosensitivity measured with lymphocytes may predict the risk of acute reaction after radiotherapy. *Int. J. Radiat. Oncol. Biol. Phys.* **71**, 256–264. 10.1016/j.ijrobp.2008.01.007 (2008). 18406889 10.1016/j.ijrobp.2008.01.007

[27] Hoeller, U. et al. Individual radiosensitivity measured with lymphocytes may be used to predict the risk of fibrosis after radiotherapy for breast cancer. *Radiother Oncol.* **69**, 137–144. 10.1016/j.radonc.2003.10.001 (2003). 14643950 10.1016/j.radonc.2003.10.001

[28] Lisowska, H. et al. Enhanced chromosomal radiosensitivity in peripheral blood lymphocytes of larynx cancer patients. *Int. J. Radiat. Oncol. Biol. Phys.* **66**, 1245–1252. 10.1016/j.ijrobp.2006.07.1370 (2006). 17145539 10.1016/j.ijrobp.2006.07.1370

[29] Yoshioka, Y. et al. Treatment planning comparison of high-dose-rate brachytherapy vs. robotic and conventional stereotactic body radiotherapy for ultrahypofractionated treatment of prostate cancer. *Phys. Imaging Radiat. Oncol.* **26**, 100445. 10.1016/j.phro.2023.100445 (2023). 37197153 10.1016/j.phro.2023.100445 PMC10183665

[30] Katz, A. J., Santoro, M., Ashley, R., Diblasio, F. & Witten, M. Stereotactic body radiotherapy for organ-confined prostate cancer. *BMC Urol.* **10**, 1. 10.1186/1471-2490-10-1 (2010). 20122161 10.1186/1471-2490-10-1 PMC2831888

