# Supplementary material for: Assessment of the predictive power the radiation-induced lymphocyte apoptosis method in prostate cancer patients
Source: Sci Rep. 2025 Jan 9;15:1516. doi: 10.1038/s41598-024-81450-7 (PMC11718057; doi:10.1038/s41598-024-81450-7)
Supplement: Supplementary file 1 — Supplementary Material 1 [file 41598_2024_81450_MOESM1_ESM.docx]

| **Age (years)** | **Mean (min-max)** | 67.3 (56-77) |
| --- | --- | --- |
| **Smoking** | **No** | 42 |
|  | **Yes** | 7 |
| **T status** | **T1** | 11 |
|  | **T2** | 38 |
| **Gleason grade group** | **Group 1** | 26 |
|  | **Group 2-3** | 22 |
|  | **Group 4** | 1 |
| **iPSA (ng/ml)** | **Mean (min-max)** | 10.7 (2.8-39) |
| **Risk (d' Amico)** | **Low** | 9 |
|  | **Intermediate** | 37 |
|  | **High** | 3 |
| **Initial IPSS** | **Mean (min-max)** | 7.9 (0-27) |
| **Hormone therapy** | **No** | 28 |
|  | **Yes** | 21 |
| **Radiotherapy** | **Cyberknife** | 35 |
|  | **HDR** | 14 |

**Supplementary Table 1.** Baseline characteristics of the patients*.* (iPSA: initial Prostate Specific Antigen, IPSS: International Prostate Symptom Score.)

|  |  | **GU** | | | | **IPSS** | | | |
| --- | --- | --- | --- | --- | --- | --- | --- | --- | --- |
|  | **RILA value (%)** | **Sensitivity** | **Specificity** | **PPV** | **NPV** | **Sensitivity** | **Specificity** | **PPV** | **NPV** |
| **lower tertile** | 5.22 | 33.3 | 69.2 | 25.0 | 73.9 | 33.3 | 69.0 | 16.7 | 82.6 |
| **upper tertile** | 18.14 | 77.8 | 38.5 | 30.4 | 83.3 | 100.0 | 41.4 | 26.1 | 100 |
| **max. Youden** | 16.78 | 77.8 | 42.3 | 31.8 | 84.6 | 100.0 | 44.8 | 27.3 | 100 |

**Supplementary Table 2.** Sensitivity and specificity values of different RILA cut-off values in the CyberKnife treated group. (GU: genitourinary side effects, IPSS: International Prostate Symptom Score, PPV: positive predictive value, NPV: negative predictive value.)

|  | **Before therapy** | **Directly after therapy** | **3 months** | **6 months** | **9 months** | **12 months** | **24 months** | **36 months** | **48 months** | **60 months** |
| --- | --- | --- | --- | --- | --- | --- | --- | --- | --- | --- |
| **Acute GU side effects** | chrom. br. |  | chrom. br. |  |  |  |  |  |  |  |
| **Acute GI side effects** |  |  |  |  |  |  |  |  | chrom. br. |  |
| **Cumulative late GU side effects** |  |  |  |  |  |  |  |  |  | total a. |
| **Cumulative late GI side effects** |  |  |  |  |  |  |  |  |  |  |
| **Acute IPSS score** | chrom. br. |  |  |  |  |  |  |  |  |  |
| **Acute QoL score** |  |  |  |  |  |  | dic., total a. |  |  |  |
| **Late cumulative IPSS increase** |  |  |  |  |  |  |  |  |  |  |
| **Late cumulative QoL increase** |  |  |  |  |  |  |  |  |  |  |
| **Late cumulative IPSS score** | ab.cell |  |  |  |  |  |  |  |  |  |
| **Late cumulative QoL score** |  |  |  |  |  |  |  |  |  |  |
| **RILA value (%)** |  |  | chrom. br. |  |  |  |  |  |  |  |

**Supplementary Table 3.** Significant correlations between chromosome aberrations, side effects and RILA values in CyberKnife treated group over time. (GU: genitourinary side effects, GI: gastrointestinal side effects, IPSS: International Prostate Symptom Score, QoL: Quality of life, chrom. br.: chromatid breaks, total a.: total aberrations, dic.: dicentric + ring chromosomes, ab.cell: aberrant cells.)

|  | **Acute GU side effects** | **Cumulative late GU side effects** | **Acute IPSS score** | **Acute QoL score** | **Late cumulative IPSS increase** | **Late cumulative QoL increase** | **Late cumulative IPSS score** | **Late cumulative QoL score** |
| --- | --- | --- | --- | --- | --- | --- | --- | --- |
| **Model p** | 0.038 | <0.001 | ns | ns | <0.0001 | 0.003 | <0.001 | 0.021 |
| **adjusted R2 (%)** | 14.5 | 56.4 | 0.0 | 0.0 | 68.0 | 37.2 | 40.8 | 18.3 |
| **RILA value (%)** |  |  |  |  | 0.041 |  |  |  |
| **Smoking (yes/no)** | 0.038 |  |  |  | <0.0001 |  | <0.001 |  |
| **Hormone therapy (yes/no)** |  |  |  |  |  | 0.008 |  | 0.021 |
| **Age** |  | 0.007 |  |  |  |  |  |  |
| **Risk (d'Amico)** |  | 0.005 |  |  |  |  |  |  |
| **Initial questionnaire score** |  |  |  |  | 0.007 | 0.008 |  |  |
| **V_10Gy_ (cm^3^)** |  | <0.0001 |  |  |  |  |  |  |
| **Total aberrations (directly after RT)** |  |  |  |  |  |  |  |  |

**Supplementary Table 4.** Multivariate regression models in the group treated with CyberKnife. (GU: genitourinary side effects, GI: gastrointestinal side effects, IPSS: International Prostate Symptom Score, QoL: Quality of life, RT: radiotherapy.)
